# A Bilateral Tessier Number 4 and 5 Facial Cleft and Surgical Strategy: A Case Report

**Published:** 2013-10

**Authors:** Shahin AbdollahiFakhim, Nikzad Shahidi, Gholamreza Bayazian

**Affiliations:** 1 *Department of Otolaryngology Head and Neck Surgery, Tabriz University of Medical Sciences, Tabriz, Iran.*

**Keywords:** Cleft lip, Facial anomaly, Facial clefts

## Abstract

**Introduction:**

Tessier facial cleft is among the rarest facial clefts reported in literatures and there are many issues arguing about its multidisciplinary repairing techniques. Tessier number 4 and 5 are extremely rare facial anomalies. There are few literatures describing these clefts and their surgical modalities. Number 5 Tessier cleft begins medial to oral commissure in the upper lip and extends superiorly as a groove through the cheek and ends at the middle third of lower eyelid. Bonny involvement consists of alveolar ridge, maxillary bone lateral to infra orbital foramen and orbits lower rim and floor. Number 4 Tessier facial cleft begins between cupid bow and oral commissure; skirting the nose and pass through cheek and lateral to lacrimal duct. Bonny involvement consists of alveolar ridge, maxillary bone medial to infra orbital foramen and orbital rim and floor.

**Case Report:**

This paper represents a patient with bilateral number 4 and 5 Tessier cleft lip with unilateral complete cleft palate and surgical approach on her.

**Conclusion:**

We recommended early repair using autogenously tissues and minimal discarding healthy tissues as possible.

## Introduction

Mostly, cleft lips involve lip with or without nose and alveolar ridge; but, facial clefts not only can involve these structures but also can split the bones and skin or make some anomalous facial features.

Tessier facial clefts involve mouth, maxilla, eyes, nose, and forehead; and may extendto viserocranium and neurocranium too. These clefts are numbered from 0 to 14, representing the extension of the cleft. These clefts can be described as oro-occular cleft and fronto-nasal dysplasia (1).

Tessier clefts have well defined positions and have definite axes. Clefts are evaluated corresponding to the mouth and eyes position. Clefts involve soft tissue and skeletal structures,including the midline to infra orbital foramen. Frequently, soft tissue is involved from infraorbital foramen to temporal bone. Skeletal defects are prominent, with exception for associated ear anomalies. These clefts do not course along main vessels and do not imply absence of main nerve and vessel trunks in the area that the cleft has appeared (2).

Clefts above upper eye lids are defined as cranial; but, clefts below lower eye lids are classified as facial ones (2).

Silva R. et al (2008), presented six patients with Tessier No.5 cleft, treated in combined centers, two patients had bilateral and four had unilateral clefts. Three of the patients had associated No.4 cleft and one patient had an associated No.3 cleft. This paper has the largest series even represented for surgery in patients with Tessier No. 5 cleft (3).

## Case Report

In 2010, a 6-month-old girl with bilateral Tessier facial cleft referred to department of pediatric otolaryngology-head and neck surgery of Tabriz-Iran children hospital. First, a thorough investigation for possibility of associated syndromes and anomalies was conducted. The consultation of pediatrician, neurologist, optometrist and cardiologist did not define any associated anomaly describing a known syndrome. All neuroimaging, hearing studies and urogenital evaluations were normal.

**Fig 1:**
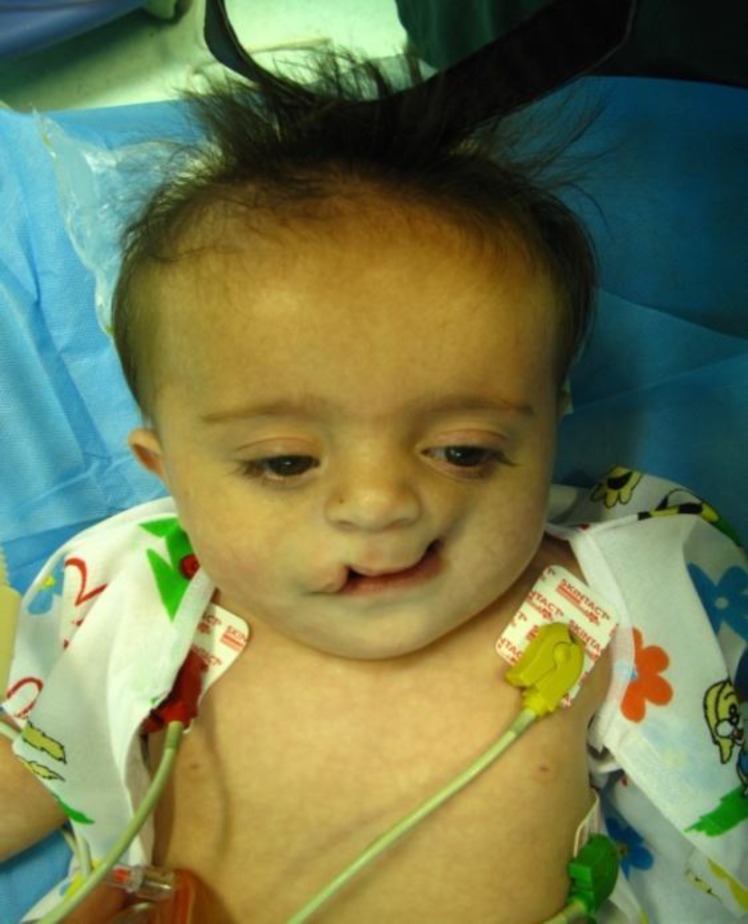
Patient with tessier cleft no. 4 and 5

She had a left side No.5 Tessier facial cleft, originating in about 10 mm medial to left commissure vermilion extending upward and laterally, making a groove on the cheek and ending at the middle third of lower eye lid, without evidence of ectropion. The medial cantus was properly in its place, but the lateral cantus was lower compared with the right side. Alveolar ridge was involved in association with unilateral complete cleft palate in the same side. Maxilla was split and rotated three dimensionally into lateral, inferior and posterior. Premaxilla was in its proper place; but, lateral alveolar ridge was rotated in the same manner. On her right side, a very mild No.4 Tessier facial cleft started lateral to phylteral column extending superiorly and lateral to right nasal alar, mildly involving alveolar ridge. No involvement of primary and secondary palate was defined (Fig.1). 

**Fig 2:**
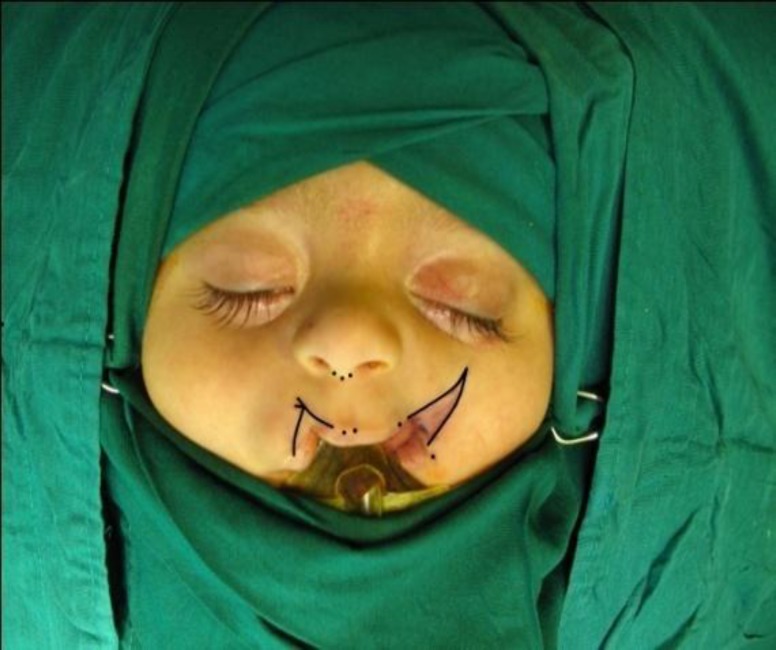
Preoperative marking for lip correction is performed

On the left side, the scar like soft tissue was marked from its high point to vermilion, sparing the commissure. Incisions were made through skin and underlying soft tissue to just above the periosteum. Bonny cleft was lateral to infra orbital foramen, therefore, incision over periosteum and dissection in a sub periosteal plane was made. Soft tissue dissection was made in two planes, one under dermal fat and one in subcutaneous plane, up to two millimeters each side of the incision. All soft tissue and periosteum were drawn medially to obscure the bonny cleft as much as possible and infraorbital neurovascular bundle were preserved laterally. Orbicularis oris muscle dissected bilaterally, preserving the zygomatic complex muscles and sutured to each other. Wet and dry mucosa sutured to each other after reconstructing advancement flaps medially and laterally, using two uni limb Z plasties bilaterally (Fig. 2).

 On the right side, the same procedure was conducted, which included scar excision, advancing subperiosteal flaps, muscle dissection, reconstructing advancement flaps medially and laterally and one uni limb Z plasty laterally, in conjunction with a back-cut in its upper end to elongate the lateral arm of the incision. Palatoplasty was postponed until the patiant’s10 months of age.

**Fig 3:**
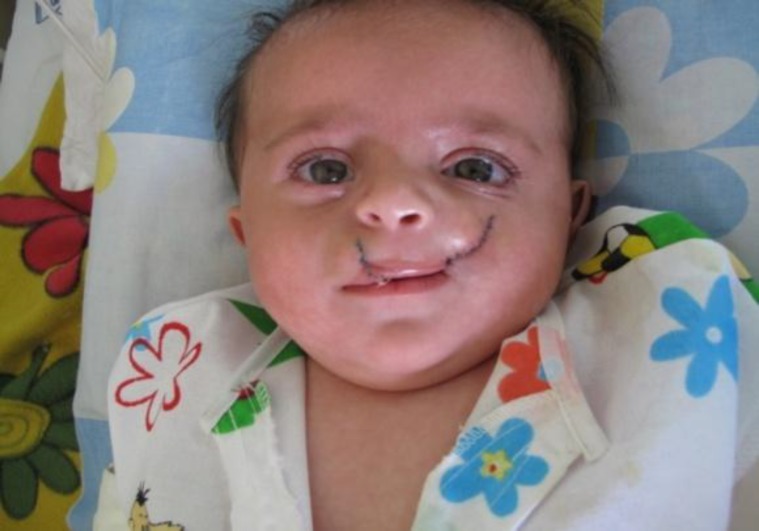
Posoperative result of lip repair

Orbicularis oris function after operation was observed and it was satisfying when the patient cried or laughed. The function of Zygomatic muscles was preserved (Fig. 3).

## Discussion

The prevalence of these groups of facial clefts is less than 0.25% among all facial clefts. Facial clefts may be associated with some congenital anomalies (5). The American Association of Cleft Palate Rehabilitation has classified the Tessier No.5 facial cleft as an orofacial canthal cleft. Boo-Chai et al (1970) called No.5 Tessier cleft as type II oro- ocular cleft (7). Karfik et al (1966) explained this, as a true oblique cleft (6), and van der Meulen et al (1985) termed this as lateral maxillary dysplasia (8). Mishra R.K. et al (2009) described their technique to fix No.3 and No.4 Tessier clefts in seven patients, with age range 1.5 to 21 years (4).

It is difficult to explain embryology of the oblique facial clefts by the known processes of craniofacial fusion. The Tessier No.5 cleft corresponds to no known embryologic grooves or plane of mesenchymally supported epithelium. The cause of these clefts may be a primary stop of development, a neurovascular insufficiency or necrosis, or tears in the developing maxillary process (3). Recent experiments suggest that these malformations are caused by a combination of directly tethered tissue migration (such as amniotic bands) and increased local pressure that produce cellular ischemia. 

## Conclusion

We present the surgical result in a 6-month-old girl with Tessier No.4 and No.5 facial cleft. We recommended early repair using autogenously tissues and as minimal disposal of the healthy tissues as possible. Early rehabilitation with massage and physiotherapy are also recommended.
